# Diastolic prolongation of forward flow in branch pulmonary artery stenosis

**DOI:** 10.1186/1532-429X-14-S1-P140

**Published:** 2012-02-01

**Authors:** Sylvia S Chen, Philip J Kilner

**Affiliations:** 1 CMR Unit, Royal Brompton Hospital, London, UK; 2 Imperial College, London, UK

## Summary

Diastolic prolongation of forward flow, or diastolic tail is present in unilateral branch pulmonary artery stenosis in patients after the arterial switch operation for transposition of the great arteries and after repair for tetralogy of Fallot. Interestingly, as a result of a diastolic tail, flow through the stenosed artery is comparable to that in the non-stenosed artery and therefore the impact of stenosis may be relieved.

## Background

Diastolic prolongation of forward flow, or diastolic tail, is known as an indicator of the hemodynamic impact of aortic coarctation or recoarctation. We have observed similarly tailed flow curves in unilaterally stenosed branch pulmonary arteries (PA) of patients with transposition of the great arteries corrected by arterial switch (TGA-AS) and after repair of tetralogy of Fallot (rToF). We are not aware that this has been described before. We aimed to improve understanding of the nature and possible clinical implications of this pathophysiological finding.

## Methods

**Figure 1:**
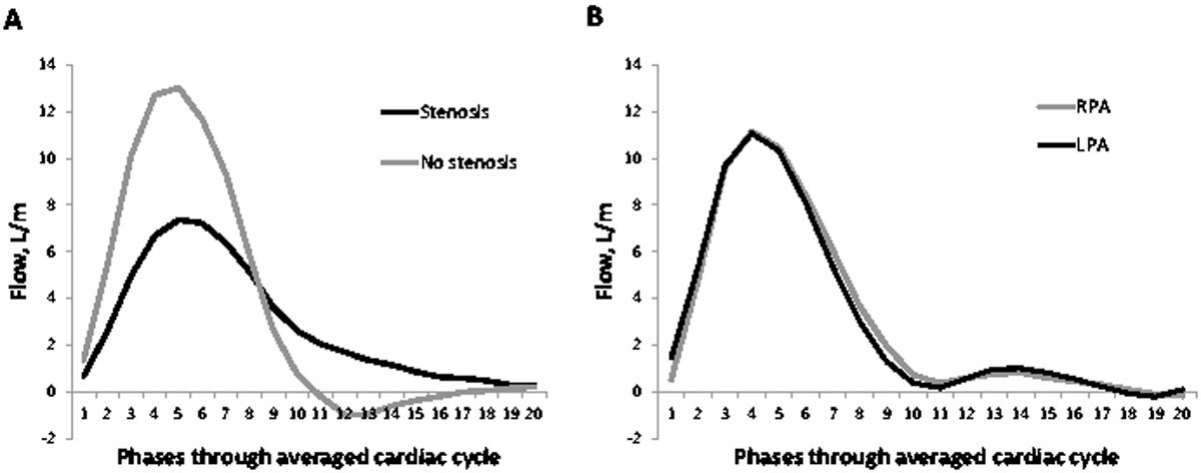
Averaged branch pulmonary artery flow curves of patients (A) and volunteers (B). In the patient group, systolic flow was lower and there was diastolic prolongation of forward flow in the stenosed relative to unstenosed branch. There is regurgitant flow from the unstenosed branch, as indicated by negative flow in diastole. In normal subjects, peak flow was equal in both branch arteries. Neither significant regurgitation nor a diastolic tail was seen in volunteers. RPA: right pulmonary artery; LPA: left pulmonary artery

Our clinical cardiovascular magnetic resonance (CMR) reports on TGA-AS and rTOF patients of the last five years (200 and 350, respectively) were searched for the above finding. It had been noticed from the appearances of cine images and through-plane velocity acquisitions aligned with or transecting, respectively, the right and left PAs. Diastolic tail was confirmed by clear discrepancy between the curves of stenosed and non-stenosed PA flow (Figure 1A). CMRtools was used to measure the volume of flow through each branch PA. The branch PA flow curves of 10 healthy volunteers aged 28±5 years, 6 male, were studied for comparison.

## Results

Ten patients, 7 TGA-AS and 3 rToF, aged (20±8 years, 8 male) were found with a diastolic tail in one or other PA. All had at least moderate branch PA stenosis, 7 on the right and 3 on the left. No patient had more than trivial pulmonary valve regurgitation. No other haemodynamically significant lesion was found. The minimum diameter of the stenosed artery was significantly smaller than the non-stenosed artery, 5.1±2.1mm vs 13.7±5.0mm, p<0.001 but, surprisingly, volumes of flow through the respective arteries were comparable, 3.0±1.5L/min on the stenosed side vs 3.4±2.2L/min, p=0.6. Flow curves through the stenosed and the non-stenosed arteries were averaged for all patients and compared with those averaged from controls (Figure 1). A diastolic tail curve was present only in stenosed arteries of patients. In spite of near competent valves, early diastolic regurgitant flow was seen from the non-stenosed PAs of patients (Figure 1A), regurgitation fraction 16±9% vs 0.2±0.3% from stenosed arteries, p<0.001. No significant regurgitation was observed in the right or left PAs of volunteers (regurgitation fraction 0.9±0.2% and 1.9±0.1% respectively); although there was slight mid diastolic forward flow (Figure 1B), attributable to proximal PA compliance.

## Conclusions

In the presence of a competent pulmonary valve, flow through a stenosed branch PA, although limited in systole, may carry on into diastole, delivered by the compliance of contralateral PA and pulmonary trunk. This may alleviate the impact of unilateral stenosis.

## Funding

There is no funding support for this study.

